# Experimental Research on Fatigue Performance of Reinforced Concrete T-Shaped Beams under Corrosion–Fatigue Coupling Action

**DOI:** 10.3390/ma16031257

**Published:** 2023-02-01

**Authors:** Tian Zhang, Xuefan Zhang, Pengfei Li, Haijiang Li, Xiaofei Li, Yunfeng Zou

**Affiliations:** 1 Transportation Engineering College, Dalian Maritime University, Dalian 116026, China; 2 Research Institute of Highway Ministry of Transport, Beijing 100088, China; 3 Cardiff School of Engineering, Cardiff University, Cardiff CF24 3AA, UK; 4 School of Civil Engineering, Central South University, Changsha 410075, China; 5 National Engineering Research Center of High-Speed Railway Construction Technology, Changsha 410075, China

**Keywords:** corrosion–fatigue coupling device, accelerated corrosion of reinforcement, reinforced concrete T-beam, corrosion-fatigue coupling test, simple fatigue test, fatigue life analysis

## Abstract

Highway bridges in coastal areas are seriously affected by the marine environment, while most of the existing test methods for bridge-reinforced concrete beams considering both corrosion and fatigue factors are carried out in an alternating manner, which cannot reflect the actual service conditions of the bridge structure. This paper focuses on an experimental study of the coupled influence of reinforcement corrosion and fatigue loading in reinforced concrete T-shaped beams. A novel loading test device that can realize the corrosion–fatigue coupling effect is designed, and then six reinforced concrete T-shaped beams are fabricated and tested. For the corrosion–fatigue coupling test beams, the variation law of beam cracks, failure modes, steel strain development law, load-deflection relationship, and fatigue life are analyzed and compared with that of the simple fatigue test beams. The test results show that the cracks of the test beam develop continuously with the fatigue loading times under the corrosion–fatigue coupling environment. The fatigue failure modes are all brittle fractures of the main steel bars, which present the shape of uneven oblique section tearing. The new testing device and approach can provide direct insights into the interaction of reinforcement corrosion and cyclic loading on the fatigue behavior of T-shaped RC beams, which can be further used to understand the long-term performance of bridge structures under complex marine environments.

## 1. Introduction

The long-term service performance of highway bridges in coastal areas, especially cross-sea bridges, is affected by the marine environment. The corrosion of steel bars in reinforced concrete bridges caused by chloride ion penetration or carbonation is very serious. Especially in recent years, in addition to the influence of the marine environment and with the rapid increase in traffic flow, the fatigue stress amplitude of the load also increases simultaneously. The combined effect of these two factors accelerates the deterioration of the fatigue performance of reinforced concrete bridges. Therefore, it is of great significance to carry out research on the performance of reinforced concrete beams under the coupled action of reinforcement corrosion caused by chloride or carbonation attack and fatigue by vehicle traffic.

Due to the limited test conditions, most of the test methods of reinforced concrete beams considering both corrosion and fatigue factors in existing related literature are carried out in an alternating manner. However, the alternating tests affected by corrosion and fatigue do not meet the actual service conditions of the bridge structure. Xu [1] found that the fatigue load obviously accelerated the corrosion of steel bars in concrete members because the cyclic load may lead to microcracks in the members, which may cause the easier ingress of chlorides through the concrete, thus touching off corrosion. On the other hand, the corrosion of steel bars obviously reduces the fatigue life of the concrete member [2]; even rebar corrosion has a significant detrimental effect on the fatigue performance of RC beams [3]. Therefore, the performance degradation of reinforced concrete beams under the corrosion–fatigue interaction is not a simple superposition of the two effects but a process of mutual promotion [4].

The interaction between fatigue and corrosion of reinforcement in reinforced concrete members has been researched in recent years; nevertheless, this field is still in need of further exploration. Some researchers focus on the corrosion behavior of steel bars and the influence of reinforcement corrosion on the mechanical properties of structures [5,6,7,8]. Some researchers have also carried out research on the connection performance under the condition of reinforcement corrosion [9,10] or on the bond behavior between concrete and corroded steel bars under reciprocating loading [11,12,13]. The influence of both sustained loading and corrosion on the performance of reinforced concrete beams is also studied [14,15]. Other studies focus on the life prediction of concrete structures exposed to a chloride environment, including precast concrete structures [16] and prestressed concrete beams [17,18], where some theoretical analysis models considering corrosion were proposed. An actual case of the failure of a reinforced concrete bridge due to marine environment corrosion was investigated by corrosion mass loss and mechanical testing in the laboratory [19]. Meanwhile, a lot of studies are based on experimental analysis of reinforced concrete beams subjected to corrosion and fatigue to evaluate the fatigue performance or predict the fatigue life [3,20,21,22,23,24,25,26,27,28,29,30]; usually, these tests deal with either the separate effect of corrosion and fatigue loading, in which corrosion and fatigue loading did not coexist simultaneously or the coupled effects of corrosion and sustain loading where loads remained constant during the tests. Only a few studies [23,25,27] have considered and realized the coupled effects of corrosion and fatigue loading in a laboratory experiment, and these limited research studies are basically for rectangular RC beams.

This paper focuses on an experimental study of the coupled influence of reinforcement corrosion and fatigue loading in reinforced concrete T-shaped beams. Firstly, the corrosion–fatigue coupled loading device is designed. Then six T-shaped beams with the same cross-section of 500 × 450 mm and a length of 3000 mm are cast. As a reference, three beams are only fatigue loaded but not corroded; the other three beams are subjected to reinforcement corrosion by the electrochemical accelerated corrosion method under simultaneous fatigue load. Furthermore, the performance of T-shaped RC beams is analyzed, including the beam crack development law, reinforcement strain change, load-deflection curve, fatigue life, and failure modes. Finally, the conclusions are summarized to provide insights into the interaction of reinforcement corrosion and cyclic loading for the fatigue behavior of T-shaped RC beams.

## 2. Corrosion–Fatigue Coupling Loading Devices for Reinforcement Concrete Beams

### 2.1. Test Method for Corrosion–Fatigue Interaction

(1) Accelerated corrosion test of steel bars

a. Impressed current method

Accelerated corrosion of steel bars by the impressed current method is the most widely used in corrosion tests of reinforced concrete structures. This technology is based on the principle of electrolysis; that is, the steel bar is connected to the positive terminal of the DC power supply, and the stainless-steel wire or plate is connected to the negative terminal. Then reinforcement corrosion is caused by applying an electrochemical potential between the anode and a cathode. The potential is varied to guarantee the current density is constant.

A typical device for reinforcement corrosion by impressed current method consists of a DC power supply, a data logger, a tank containing NaCl solution, the stainless-steel plate or wire partially immersed in NaCl solution connected to the negative terminal of the DC power supply, and the steel bars in reinforcement concrete structures connected to the positive terminal [31]. Electrochemical reactions take place once the voltage is applied to the device. The data logger is applied to record the current intensity.

b. Artificial climate environment method

The impressed current method mentioned above was developed based on electrochemical principles, using a DC power supply and immersing the reinforcement concrete beam in NaCl solution. In order to better simulate the natural corrosion process in a short period of time and develop a reasonable model of steel bars corrosion in real reinforcement concrete structures, it is considered that in the natural environment, the main factors affecting the corrosion of reinforced concrete structures are the humidity, temperature, and oxygen. The artificial climate environment method is often developed by considering these factors, and it is expected to create a favorable environment for accelerated corrosion. Accelerating the corrosion process by splashing, spraying salt water, and changing climate conditions without applying an impressed current, it is possible to reproduce the natural corrosion process of reinforcement. Typically, artificial climate environment technology is performed by placing the sample in an environmental chamber where the temperature and relative humidity can be monitored, and nozzles, even loading fixtures, are adjustable. Yuan et al. [32] and Li [33] utilized this method to research the corrosion of reinforced concrete beam subjected to drying–wetting cycles.

(2) Alternating tests of fatigue and corrosion

The experimental beam is cyclically loaded in the air for a certain number of times and then unloaded and removed to a tank filled with NaCl solution so that the tensile area of the experimental beam is immersed in NaCl solution for reinforcement corrosion. This process can also be accelerated by the applied current. After the experimental beam is corroded for a certain time, it is dried treatment by a Yuba lamp. The alternating action of a corrosive environment and fatigue load is realized by several cycles. In this way, the fatigue–corrosion interaction is studied.

(3) Fatigue–corrosion coupling tests in artificial climate environment

The experimental beam is placed in the artificial climate chamber, and the fatigue load is applied to realize chemical corrosion under the action of fatigue. This method is simple and easy to operate, and pitting, and uneven corrosion distribution on the surface of steel bars can be obtained, which is very close to the natural corrosion shape of steel bars. Although the corrosion–fatigue coupling tests can be conducted by artificial simulated climate conditions, this method is only applicable to the experiment of small-sized members. The experiment is time-consuming and costly.

(4) Fatigue–corrosion coupling tests accelerated by impressed current

According to the development of the fatigue–corrosion coupling test method and combining the advantages of the impressed current method and artificial climate environment method, a coupling test device is designed to apply fatigue loads while achieving accelerated corrosion of steel bars considering the factors of test requirements, time and cost in this paper. The specific scheme is to install fixed water spraying facilities around the test beam so that the beam can be continuously infiltrated by the NaCl solution to simulate the marine environment. Meanwhile, multi-circle stainless-steel wire is fixed to the outer surface of the test beam. The rebar head exposed in the test beam is connected to the positive electrode of the DC power supply, and the negative electrode is connected to the stainless-steel wire on the test beam. The current circuit is generated between the steel bar and the stainless-steel wire to complete the impressed current accelerated corrosion of the steel bar. At the same time, the hydraulic servo loading test machine is used to carry out fatigue loading on the test beam.

### 2.2. Coupling Test Device of Corrosion Environment and Cyclic Loads

#### 2.2.1. Test Device Design

**Figure 1:**
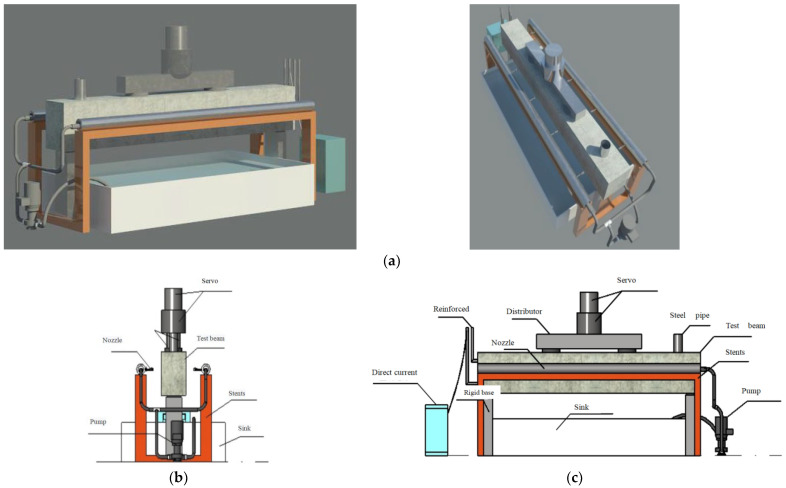
Coupling test device considering both corrosion environment and cyclic loads. (**a**) Design sketch. (**b**) Side view. (**c**) Front view.

According to the test method described above, a new spray test and loading device is designed, which can be used to simulate the corrosion environment and the cyclic loading simultaneously. The design diagram of the proposed test device is shown in Figure 1.

#### 2.2.2. Construction Process of the Coupling Test Device

A corrosion–fatigue coupling test device is formed by assembling the spray facilities, the reinforcement accelerated corrosion device, and the loading equipment. Through the combination of a water pump, steel frame, sprinkler, timing power switch, and other devices, intermittent spraying is carried out. The spraying is stopped every half an hour and intermittently for 5 min to keep the beam continuously wet, and the stainless-steel wire and the rebar to be corroded are connected to the DC power supply to generate a current circuit accelerating the reinforcement corrosion. The loading device adopts a hydraulic servo testing machine, which can effectively control the fatigue loading amplitude and loading frequency. By controlling the wetted area of the test beam, using sponge to prevent the NaCl solution from splashing, and matching with the circulating water tank, the continuous circulation of NaCl solution in the water tank is ensured, which strengthens the protection of the loading device.

**Figure 2:**
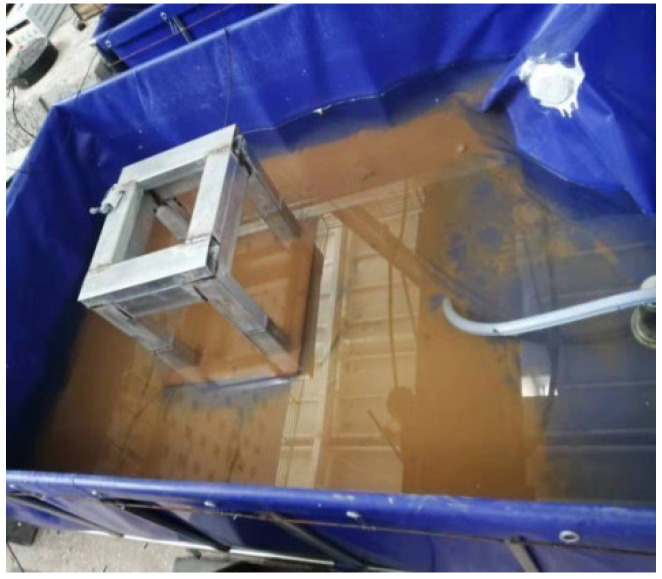
Water tank.

**Figure 3:**
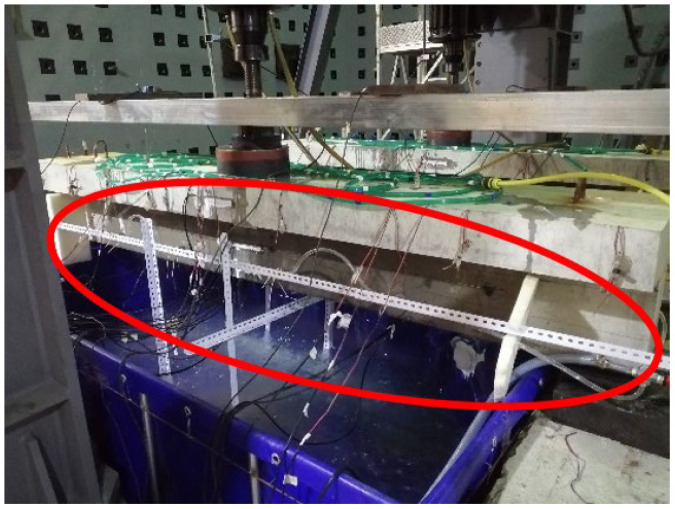
Frame supporting spraying facilities.

**Figure 4:**
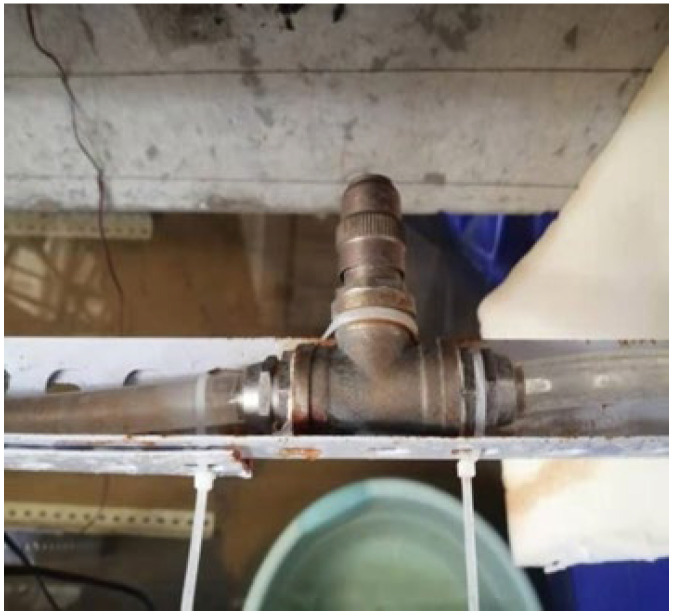
Spray nozzle.

**Figure 5:**
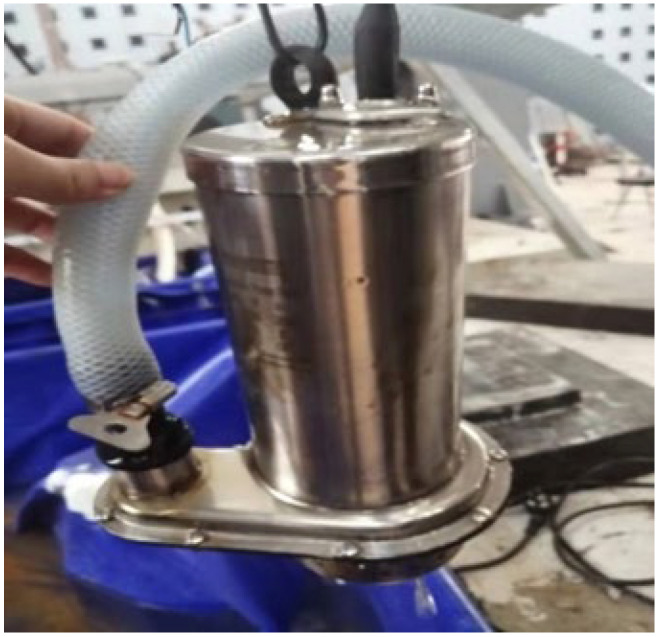
Stainless-steel water pump.

**Figure 6:**
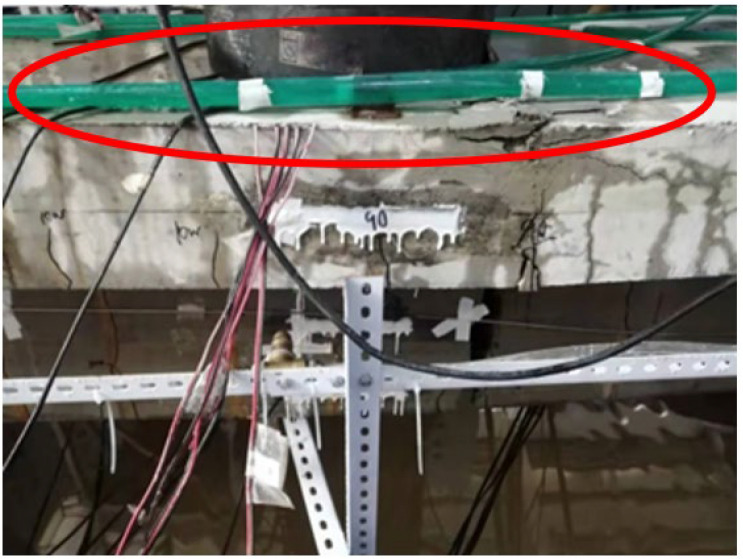
Drip irrigation pipe.

**Figure 7:**
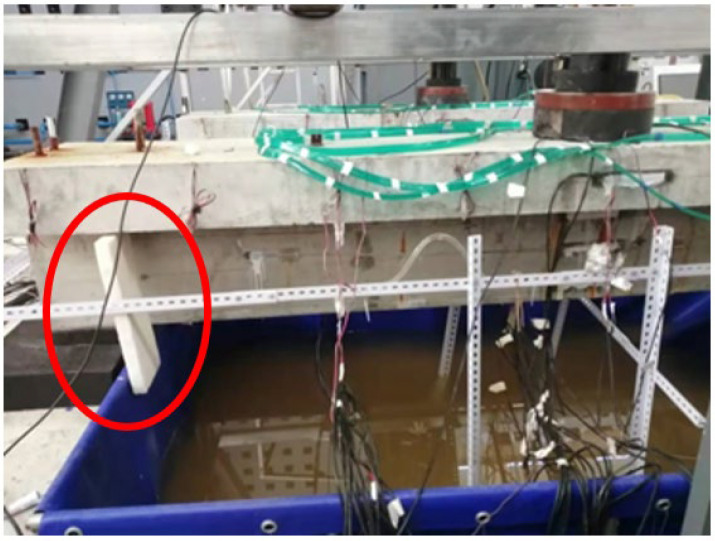
Sponge.

**Figure 8:**
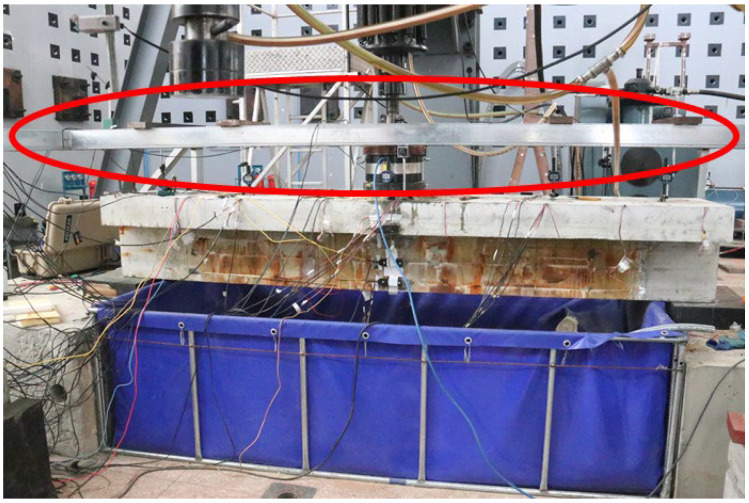
Square steel frame.

The detailed construction process is as follows:

(1) A water tank, as shown in Figure 2, is built at the bottom of the test beam to store the NaCl solution for the test.
(2) A steel frame, as shown in Figure 3, is built around the test beam. Four spray nozzles are placed at equal intervals on both sides of the test beam and are stably mounted on the frame through water pipes, as shown in Figure 4.
(3) Considering the corrosion of the water pump itself, the test device uses a stainless-steel submersible pump, as shown in Figure 5. The submersible pump is placed in the water tank, and the water level just exceeds the height of the water pump. By connecting the water pump to the water pipe, the nozzles can stably spray NaCl solution in the water tank on the side of the test beam, and the excess NaCl solution flows back to the water tank for recycling.

(4) The top surface of the test beam is watered with a drip irrigation pipe, as shown in Figure 6. The drip irrigation pipe is placed on the top surface of the test beam. Through its connection with the water pump, the NaCl solution in the drip irrigation pipe is stable and continuous, then the top surface and flange side of the test beam are wetted.
(5) In order to prevent the nozzles on both sides of the test beam from splashing NaCl solution out of the water tank, sponges are installed and fixed on both ends of the test beam, as shown in Figure 7, which can effectively absorb the splashed NaCl solution, and the flexibility of the sponges can minimize the impact on the fatigue of the test beam.

(6) Since the water tank is placed under the test beam, it is impossible to measure the deflection of the test beam by the pull wire displacement sensor under the test beam. Therefore, a square steel frame is arranged on the top of the test beam, as shown in Figure 8, and a pull wire displacement sensor is fixed on the frame to avoid damage to the pull wire sensor by water.

**Figure 9:**
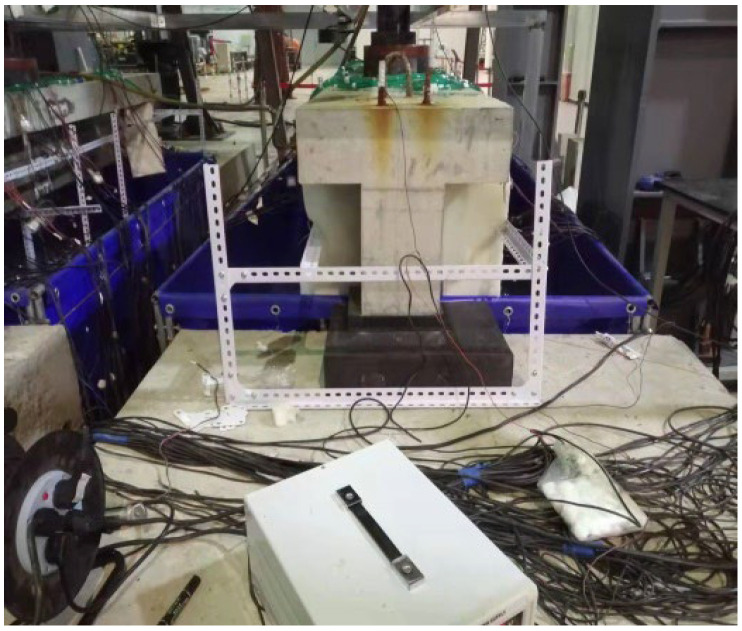
Corrosion–fatigue coupling test device.

Through the innovative design of the test device, the following problems are mainly solved: fatigue loading and reinforcement corrosion are carried out simultaneously; effective simulation of spraying time and environmental effect; protection of hydraulic servo loading equipment in salt fog environment; isolation of test beam reaction device and support system from salt fog environment, etc. The physical device of the corrosion–fatigue coupling test is shown in Figure 9.

## 3. Corrosion–Fatigue Coupling Tests of Reinforced Concrete Beams

### 3.1. Design of Reinforced Concrete T-Shape Test Beam

**Figure 10:**
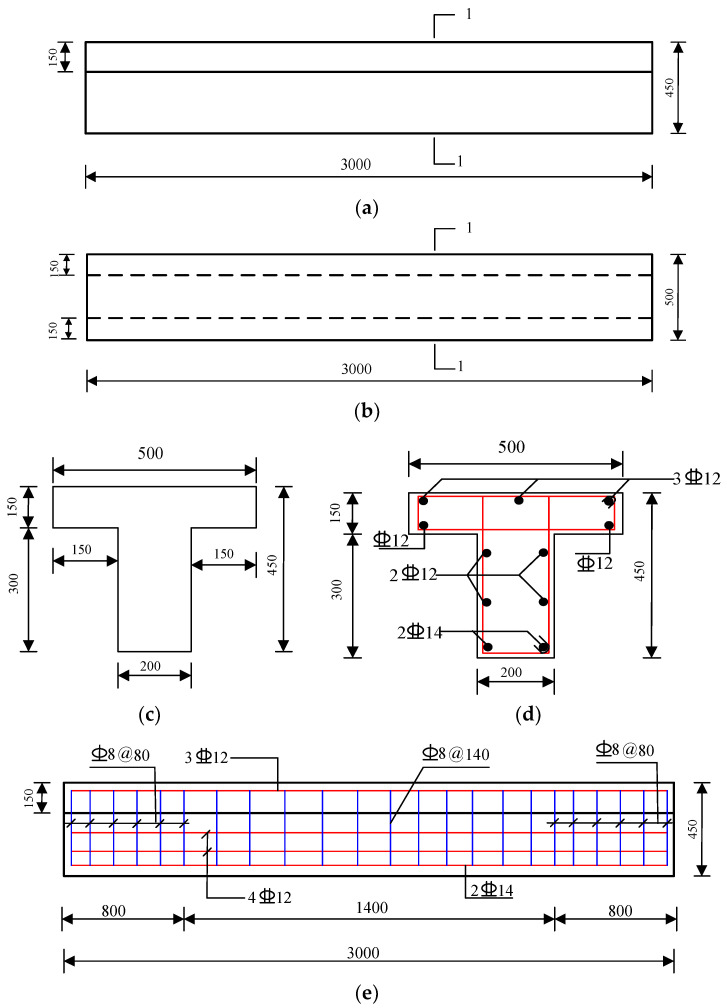
Geometry and reinforcement drawings of the test beam (mm). (**a**) Elevation. (**b**) Vertical view. (**c**) Side view. (**d**) 1-1 cross-section profile. (**e**) Longitudinal-section profile.

In the design process of these test beams, considering that the test beams in the previous fatigue tests were mostly rectangular, but the proportion of rectangular beams used in practice projects is small, so the actual highway-reinforced concrete T-beam is selected for this test, and the geometric size of the test beams is calculated by a certain proportion of the scale, and on the premise of keeping the reinforcement ratio unchanged, the reinforcement of the test beam is rearranged. The span of the test beam is 3 m, the thickness of the concrete cover is 4 cm, and the beam end constraints are simply supported type. The test beam is a reinforced concrete T-shaped beam, with the bottom width of the rib *b* = 200 mm, the top plate width *b_f_* = 500 mm, the flange height *h_f_* = 150 mm, and the total beam height *h* = 450 mm. The beam rib is provided with 6 longitudinal steel bars, and the lower part of the flange is provided with 2 longitudinal steel bars, with a total sectional area *A_s_* = 986.5 mm^2^; the upper part of the top plate is provided with 3 longitudinal steel bars, and the sectional area is *A_s_’* = 226.2 mm^2^. The test beam is poured with C50 concrete, and the concrete compressive strength *f_ck_* = 41.3 MPa and the elastic modulus *E_c_* = 39,940 MPa are measured by the material test. The longitudinal steel bar at the rib bottom of the test beam is an HRB400 rebar with a diameter of 14 mm, and the other longitudinal steel bars are HRB400 rebar with a diameter of 12 mm. The stirrup is an HRB335 rebar with a diameter of 8 mm. The geometric dimension and reinforcement drawing of the test beam are shown in Figure 10.

**Figure 11:**
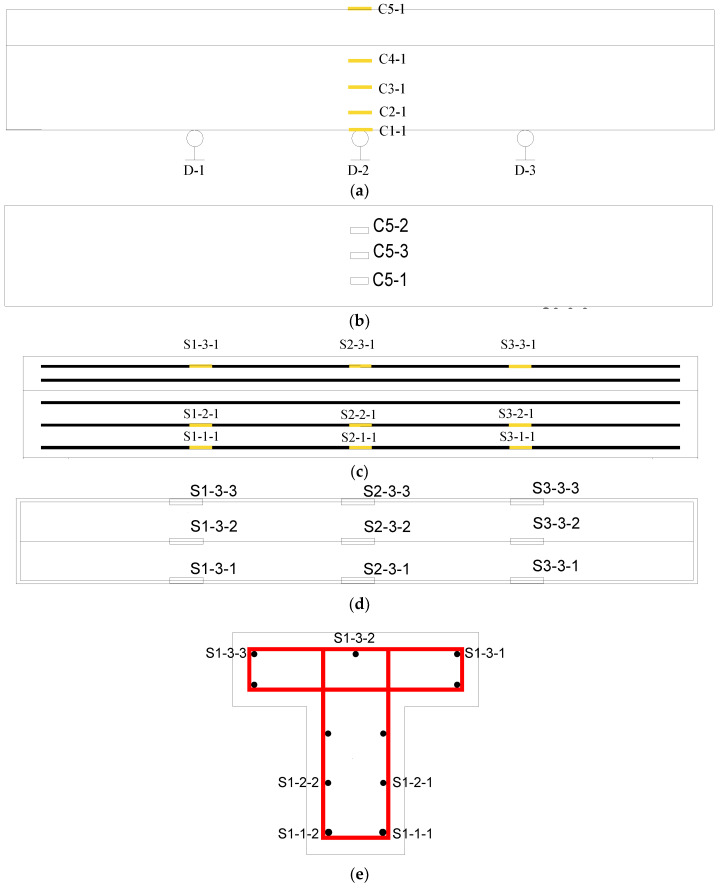
Layout of strain and displacement measuring points. (**a**) Side view of strain measuring points of concrete and displacement measuring points of the test beam. (**b**) Top view of strain measuring points of concrete. (**c**) Side view of strain measuring points of steel bars. (**d**) Top view of strain measuring points of steel bars. (**e**) Strain measuring points of steel bars in Section 1.

In order to measure the responses of the test beam during the experiment to evaluate the mechanic performance of the beam, strain gauges shall be pasted on both sides of the rib of the test beam, the top surface, and the reinforcement surface, and displacement meters shall be installed at the bottom of the test beam. The arrangement of measuring points is shown in Figure 11. The naming format of the concrete strain gauge number is CX-X, where X is a number, the first number from small to large represents the relative height changing from low to high, and the second number indicates the side direction of the concrete beam. Generally, the number 1 represents the strain gauge on the south side. For example, the concrete strain gauge at the bottom measuring point on the south side is numbered C1-1, and the corresponding strain gauge on the north side is numbered C1-2. The naming format of the steel bar strain gauge number is SX-X-X, where X is also a number, the first number represents the section number, the second number represents the relative height of the steel bar position, and the third number represents the number of the steel bar on each side. Generally, the number 1 represents the steel bar on the south side; for example, S1-1-1 represents the strain gauge on the steel bar at the south side of the bottom layer in Section 1. The number of the displacement meter is D-1, D-2, and D-3, respectively, to indicate the displacement measuring points of the left 1/4 span, the middle span, and the right 1/4 span of the beam. ‘C’ represents concrete strain, ‘S’ represents steel bar strain, and ‘D’ represents displacement.

### 3.2. Corrosion–Fatigue Coupling Test Scheme

Three different loading amplitudes are selected to carry out the corrosion–fatigue coupling tests. During the loading process, the test beam is continuously sprayed with salt solution and electrified to accelerate the corrosion of reinforcement. As a reference, three beams are only fatigue loaded but not corroded, considering the above three fatigue-loading amplitudes.

(1) Fatigue-loading amplitude

In order to simulate the effect of actual vehicle load, taking the reinforcement stress level of the actual bridge as the standard and considering the China Highway-I vehicle load [34], the upper limit of fatigue load corresponding to the test beam is 70 kN, and the lower limit of fatigue load is 20 kN. Based on this, if the proportional overload of 15% is considered, the upper limit of fatigue load is about 80 kN; if a more conservative estimation is made, the upper limit of fatigue is about 90 kN when 30% overload is applied. Therefore, according to the corresponding lower limit of fatigue load and upper limit of fatigue load, corrosion–fatigue coupling tests and pure fatigue tests are conducted. The specific cases are listed in Table 1, where ‘XP’ represents corrosion–fatigue coupling tests, and ‘P’ represents simple fatigue tests.

(2) Fatigue loading method

The hydraulic servo fatigue testing machine is used for loading. When the set cycle times (increasing by 100,000 times) are reached, the load is unloaded to zero step by step, and the test beam is left standing for 2 min. Then the residual deflection and residual strain are measured. After the record is completed, the static load shall be loaded to the lower limit of fatigue load *P*_min_, and then the load shall be loaded to the upper limit of fatigue load *P*_max_ step by step. The test result data at the upper limit of fatigue load shall also be recorded, including deflection, concrete strain, reinforcement strain, crack width, and development status. After that, the reciprocating load cycle shall be continued, and the loading frequency is 4 Hz.

The fatigue failure of the member is considered to occur when the member reaches one of the following conditions: (1) the concrete in the compression area is crushed; (2) fracture of longitudinal reinforcement; (3) a rib reinforcement (stirrup or bent reinforcement) intersecting with the critical oblique crack is broken; (4) shear compression fatigue failure of concrete.

If fatigue failure occurs when the number of cycles is less than 2 million times, the limit cycle number shall be recorded. If the cyclic loading times exceed 2 million, record the fatigue test result data at 2 million times, then apply the static load step by step until the test beam is damaged, and record the maximum load at the time of failure.

(3) Current density during accelerated corrosion

**Figure 12:**
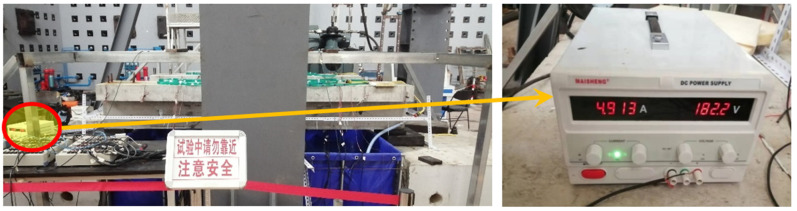
Constant current and constant voltage power supply.

In Reference [35], 133 rebars with different rust degrees were taken from the reinforced concrete members on site, among which the rebars with a corrosion rate of about 5% accounted for a relatively high proportion. Therefore, a 5% corrosion rate is taken as the final corrosion rate of the corrosion–fatigue coupling test beam in this research. Before the corrosion–fatigue tests, it was estimated that the fatigue life of the corrosion–fatigue reinforced concrete beam corresponding to China Highway-I vehicle load is more than 2 million times, by referring to the previous research result [25]. Then the theoretical corrosion rate of reinforced concrete beams can be calculated by Faraday’s electrolysis law [36], and the constant current density during accelerated corrosion is derived as 0.45 mA/cm^2^ by constant current and constant voltage power supply shown in Figure 12.

### 3.3. Description of Test Phenomenon and Test Result Analysis

#### 3.3.1. Description of the Test Phenomenon

For the corrosion–fatigue coupling tests and simple fatigue tests, the test phenomenon is summarized in Table 2.

During the fatigue loading process of reinforced concrete T-shape test beams under the coupling action of corrosion and fatigue, the concrete cracks are constantly opened and closed. When the cracks are opened, negative pressure is generated at the crack, and the salt water is sucked into the concrete. When the cracks are closed, the salt water and the corrosion products are extruded. With the increase in the number of cycles, the rust at the crack continues to accumulate, especially in the midspan and both sides of the midspan. After 200,000 to 300,000 times, it is difficult to measure the crack width due to the influence of the rust.

Both P-2 and P-3 test beams have fatigue failure before 2 million cycles, which is mainly manifested as brittle failure of bottom reinforcements. The cracks of P-2 and P-3 fatigue test beams show a three-stage development law. In the first stage, i.e., the number of cyclic loadings is less than 100,000 times, and the crack development speed is fast. During this period, the crack in the web has been basically completed. In the second stage, i.e., when the number of cycles is 100,000 times 90% of the fatigue life, it enters the stable development stage, and no new cracks are generated in the web. It further develops on the basis of the original cracks and increases the crack width, but cracks appear on the flange. In the third stage, at the last 10% of the fatigue life and near the failure state, the main crack in the mid-span extends upward, widens, and rapidly penetrates the entire section, and branch-like new cracks are generated at the top flange of the main crack until the reinforcement breaks, and other cracks tend to close gradually except the main crack.

In case of fatigue failure, the two tensile longitudinal bars at the bottom suddenly break, and the sound of the steel bar breaking can be heard during loading. At this time, the mid-span deflection of the beam rapidly drops, the jack begins to be unstressed, the upper limit and amplitude of load begin to drop, the concrete at the crack in the mid-span begins to crack and fall, the crack rapidly expands, and the second layer of steel bars at the bottom of the beam begins to bear all tensile stress, soon, the second layer of steel bars are suddenly broken, and the deflection in the mid-span rapidly decreased again. The stopper began to work, and the loading system stopped. Finally, the test beam loses its bearing capacity due to the fracture of the tensile reinforcement at the bottom two layers. At this time, the concrete in the compression area rises and falls off, the concrete at the bottom of the mid-span also falls off, and the stirrup and the longitudinal reinforcement at the bottom are exposed.

#### 3.3.2. Analysis of Fracture Development for Test Beams

For the corrosion–fatigue coupling tests and simple fatigue tests, the fracture development law of these test beams is summarized in Table 3.

(1) Corrosion–fatigue coupling test beams

a. XP-1 test beam

**Figure 13:**
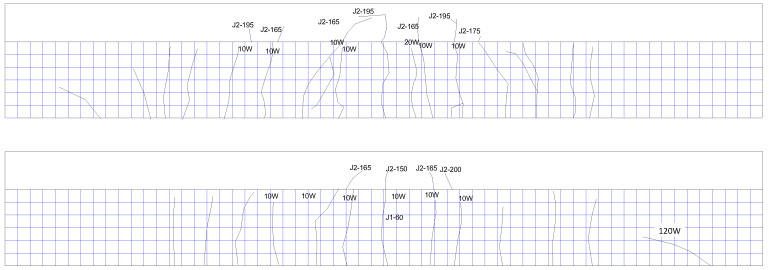
Crack distribution of XP-1 test beam during static load failure after 2 million times cyclic loading (both sides of rib).

The upper limit of the fatigue load of the XP-1 test beam is 70 kN, and there is no obvious change at the initial stage of loading. With the progress of the fatigue test, cracks begin to develop. Most of the cracks extend vertically along the beam height, which is all typical bending cracks. After two million cycles of loading, the test beam has no fatigue failure, and then the test beam is statically loaded until failure. When the static load reaches 140 kN, the test beam enters the yield stage, and when the load is greater than 160 kN, the cracks of the flange begin to appear and expand into a large number of secondary cracks at 190 kN; finally, the test beam is damaged due to crushing of concrete at the top flange when the load is 225 kN. The crack distribution is shown in Figure 13.

b. XP-2 test beam

**Figure 14:**
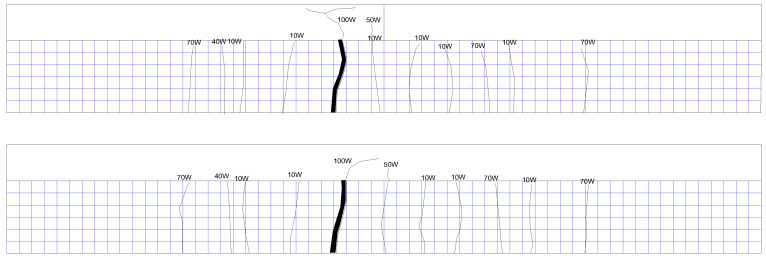
Crack distribution of XP-2 test beam at fatigue failure (both sides of rib).

The upper limit of the fatigue load of the XP-2 test beam is 80 kN, and there is no obvious change at the initial stage of loading. When the cyclic load reaches 1.01 million times, the fatigue failure of the XP-2 test beam occurs, and the specific crack distribution is shown in Figure 14.

c. XP-3 test beam

**Figure 15:**
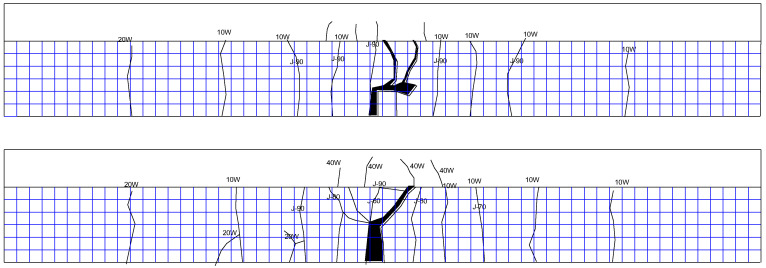
Crack distribution of XP-3 test beam at fatigue failure (both sides of rib).

The upper limit of the fatigue load of the XP-3 test beam is 90 kN, and there is no obvious change at the initial stage of loading. The fatigue failure occurred at 430,000 times cyclic loading. The crack distribution is shown in Figure 15.

(2) Simple fatigue test beams

a. P-1 test beam

**Figure 16:**
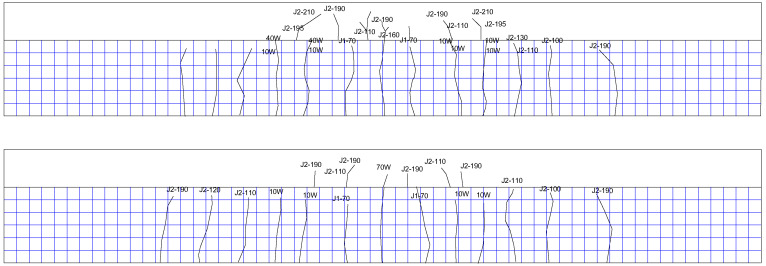
Crack distribution of P-1 test beam during static load failure after 2 million times cyclic loading (both sides of rib).

The upper limit of the fatigue load of the P-1 test beam is 70 kN, and there is no obvious change at the initial stage of loading. With the progress of the fatigue test, cracks begin to develop. Most of the cracks extend vertically along the beam height, which are all typical bending cracks. After two million times of fatigue loading, no fatigue failure occurred in the test beam. Then the static load is applied until the beam is damaged. With the increase in load, new cracks appear at both ends of the test beam at 100 kN, 110 kN, and 190 kN. When the static load reaches 110 kN, cracks appear on the flange, and from 160 kN to 210 kN, the flange cracks continue to develop. Finally, when the load is 252 kN, the test beam fails due to the crushing of concrete at the top flange, as shown in Figure 16, which is the crack distribution at the failure of the test beam.

b. P-2 test beam

**Figure 17:**
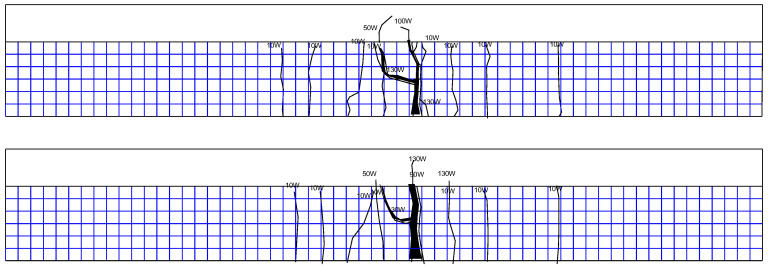
Crack distribution of P-2 test beam at fatigue failure (both sides of rib).

The upper limit of the fatigue load of the P-2 test beam is 80 kN, and there is no obvious change at the initial stage of loading. With the progress of the fatigue test, cracks begin to develop, and most of the cracks extend vertically along the beam height, which are typical bending cracks. The crack distribution of the test beam is shown in Figure 17 at fatigue failure of the P-2 test beam.

c. P-3 test beam

**Figure 18:**
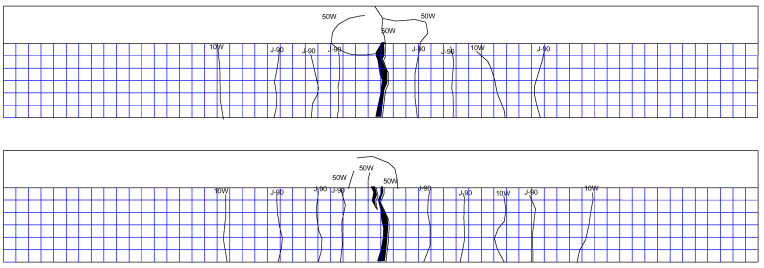
Crack distribution of P-3 test beam at fatigue failure (both sides of rib).

The upper limit of the fatigue load of the P-3 test beam is 90 kN, and there is no obvious change at the initial stage of loading. The fatigue failure occurs at 530,000 times cycle loading. The crack distribution is shown in Figure 18.

(3) Mechanism analysis of the crack development

Compared to the results of simple fatigue test beams, the number of concrete cracks in corrosion–fatigue coupling test beams is increasing considerably, and the width of cracks is larger under the same conditions. It is probably caused by the generation of expansion pressure and the presence of cracks originating from the corrosion process. Because once steel corrosion begins in the corrosion–fatigue coupling test beams, the corrosion products can expand freely until the porous zone around the steel bars is filled, and then the expansion pressure surrounding the concrete begins to develop. With the progress of corrosion, the expansion pressure can cause tensile stresses and strains in adjacent concrete. When the tensile stress at any part of the inner area reaches the tensile strength of the concrete, the corrosion-induced cracks in the concrete will occur firstly at the steel bar–concrete interface and then expand to the external surface of the cover gradually. At the same time, fatigue load will aggravate this effect, leading to an increase in the number and width of cracks.

#### 3.3.3. Failure Mode Analysis of Test Beams

(1) Failure mode

**Figure 19:**
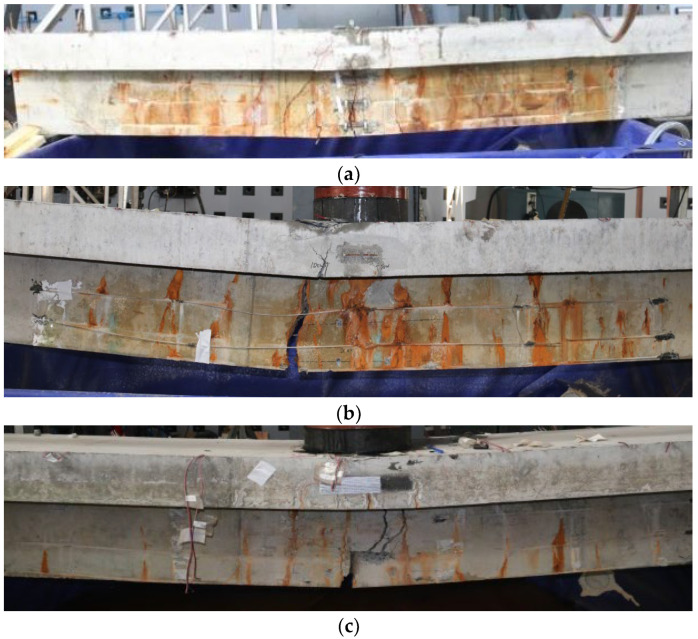
Failure mode of corrosion–fatigue coupling test beams. (**a**) XP-1 test beam. (**b**) XP-2 test beam. (**c**) XP-3 test beam.

**Figure 20:**
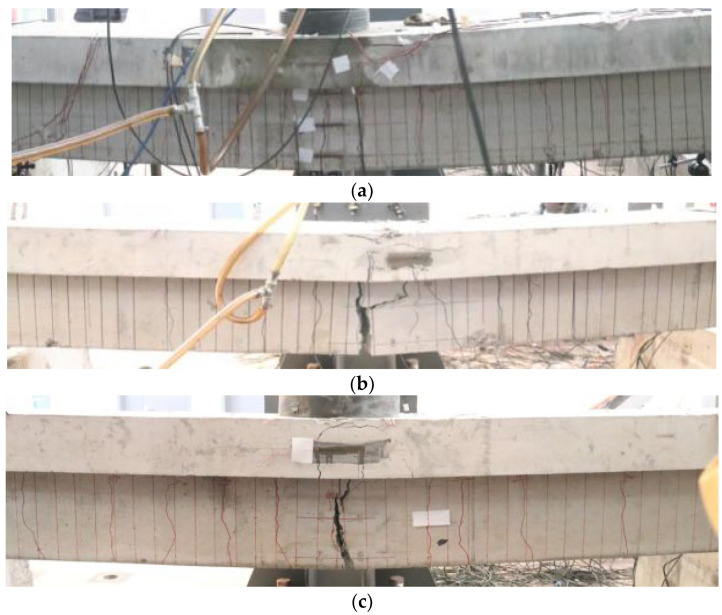
Failure mode of simple fatigue test beams. (**a**) P-1 test beam. (**b**) P-2 test beam. (**c**) P-3 test beam.

For the corrosion–fatigue coupling tests and simple fatigue tests, the failure mode of the test beams is summarized in Table 4. The failure status of each beam is shown in Figure 19 and Figure 20.

(2) Mechanism analysis of the interaction between corrosion and fatigue

According to the test results and phenomena, the basic characteristics of the corrosion–fatigue process are as follows: (1) the fatigue failure of corrosion–fatigue coupling reinforced concrete beams is due to the brittle fracture of steel bars; (2) the fatigue fracture of the corroded steel bar occurs near the position corresponding to the main crack; (3) the corrosion of steel bars shows the characteristic of a “point rust”, and the corrosion area is mainly concentrated in the crack position of the test beam; (4) the fracture of the steel bar under corrosion–fatigue interaction presents a tear inclined cross-sectional shape with uneven surface and strong graininess, which is obviously different from the smooth fracture of the steel bar in simple fatigue test beams.

Based on the existing research results and the above characteristics from the test, the interaction mechanism between corrosion and fatigue can be understood from the following aspects. Firstly, a passivation film forms on the surface of the steel bar in the high-alkaline concrete, but fatigue cracks are generated in the test beams under the fatigue stress, and the Cl^−^, O_2_, and H_2_O gradually permeate into the reinforcement surface through these cracks. Thus, the passivation film is gradually broken in the environment of chloride ions, and the exposed fresh metal belongs to the anode relative to the passivation film, which constitutes a corrosion battery and makes corrosion occur. This kind of corrosion is uneven corrosion of steel bars; that is, corrosion of the steel bar occurs mainly at the cracks of the test beam. At the same time, the corrosion products concentrated in the cracks have the rust swelling effect, which intensifies the development of cracks, further accelerates the corrosion rate, and the actual stress state of the main steel bars at the cracks becomes more complicated. In addition, the fatigue fracture of corroded steel bars originates from the generation of local cracks inside the steel bars. After the initiation of fatigue cracks of steel bars at the main cracks in the test beam, salt water is adsorbed on the surface of the steel bars and inside the cracks so that chloride ions and corrosion products are generated inside the cracks. The interaction with fatigue load accelerates the expansion of the cracks and generates oblique principal stresses. At the same time, under the action of the current, the hydrogen atoms inside the steel bar surge [38,39], resulting in hydrogen embrittlement, which reduces the internal plasticity of the reinforcement and presents an uneven oblique section tear. Therefore, the coupling effect of steel corrosion and fatigue load is not a simple superposition of the two but promotes each other, thus accelerating the damage of steel bars.

#### 3.3.4. Strain Analysis of Longitudinal Reinforcements

(1) Changing law of longitudinal reinforcement strain in test beams

**Figure 21:**
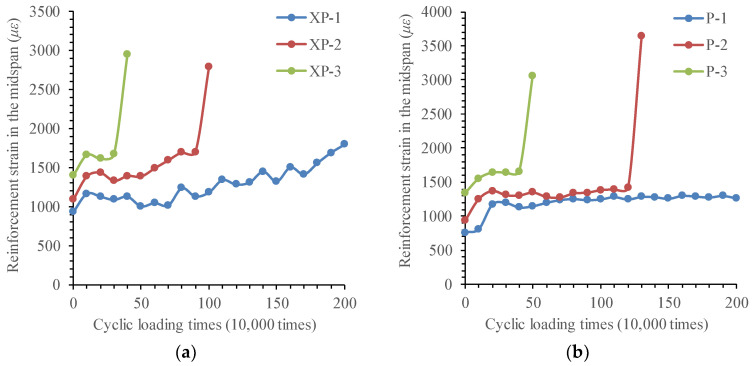
Changing curve of reinforcement strain in the midspan with cycle times. (**a**) Corrosion–fatigue coupling test beams. (**b**) Simple fatigue test beams.

In the process of cyclic loading for test beams, taking the average value of the reinforcement strain in the same section as the representative value, the changing law of the tensile strain of the bottom longitudinal reinforcement in the midspan of the test beam with the number of cyclic loadings is shown in Figure 21. It can be inferred from the figure that when the corrosion–fatigue coupling effect reaches a certain degree, it accelerates the expansion of fatigue crack, then lead to the transformation of reinforcement strain from steady state to linear growth. Judging from the time of reinforcement strain transformation of XP-1 and XP-2 test beams, corrosion–fatigue interaction plays a mutual role in promoting the expansion of fatigue crack of rebars. With the increase in fatigue stress amplitude, this promotion role becomes more obvious. Until the fatigue fracture of the reinforcement occurs, the maximum strain in the reinforcement does not reach the yield strain.

For the corrosion–fatigue coupling tests and simple fatigue tests, the variation law of strain of longitudinal reinforcements in test beams is summarized in Table 5.

(2) Mechanism analysis of the reinforcement strain development

The strain results of the reinforcement show that the longitudinal reinforcement is always in a high-stress working state under cyclic loading, and the fatigue plastic cumulative damage of the reinforcement increases rapidly. The ultimate fatigue failure of the beam is the fracture brittle failure caused by the fatigue crack development of the reinforcement, not the tensile yield of the reinforcement itself.

Compared to the results of simple fatigue test beams, the steel bars in the corrosion–fatigue coupling test beams exhibit larger longitudinal tensile strains. In the stable development stage of reinforcement strain, for all corroded beams, longitudinal tensile strains increase in a fluctuating manner with cyclic loading times, whilst for an uncorroded beam, longitudinal tensile strains fluctuate around the same value. Measured longitudinal tensile strains of the steel bars indicate that longitudinal tensile strains induced by the corrosion of steel bars are significantly larger than tensile strains due to transverse cracks from the imposed load. The effect of corrosion on the longitudinal tensile strains of steel bars may be attributed to the following three aspects: (a) the loss in the cross-section area of tensile steel bars; (b) the loss in the bond between the steel bars and concrete due to cracking; (c) secondary longitudinal strains induced by lateral tensile strains from the expansive corrosion products.

#### 3.3.5. Analysis of Deflection Change and Stiffness Degradation of Test Beams

(1) Changing the law of deflection of test beams

**Figure 22:**
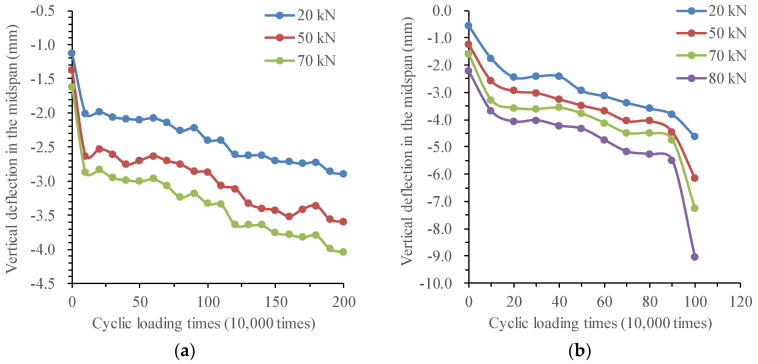

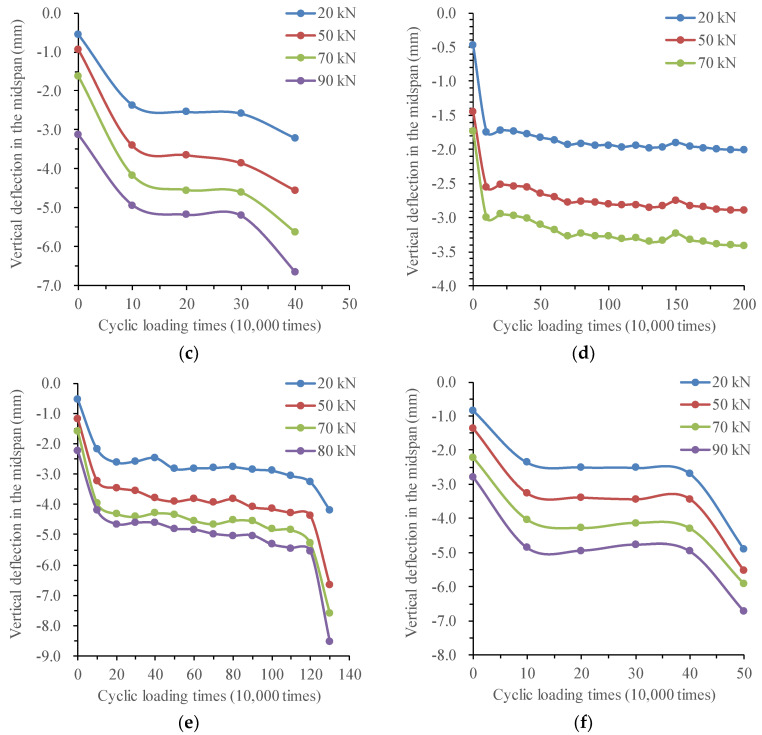
Midspan deflection of test beams with cycle times. (**a**) XP-1 test beam. (**b**) XP-2 test beam. (**c**) XP-3 test beam. (**d**) P-1 test beam. (**e**) P-2 test beam. (**f**) P-3 test beam.

When the fatigue loading times reach the multiple of 100,000 times, the static load test is carried out on the beam to discuss the effect of corrosion–fatigue interaction on the stiffness of reinforced concrete beams. The variation curve of mid-span deflection of corrosion–fatigue coupled beams XP-1, XP-2, and XP-3 with fatigue cycle times under different levels of the static load is shown in Figure 22, as well as that of different simple fatigue test beams.

For the corrosion–fatigue coupling tests and simple fatigue tests, the changing law of deflection of test beams is summarized in Table 6.

In order to quantitatively describe the stiffness degradation process of test beams, the deflection difference between the first static load and the last static load of XP-1, XP-2, and XP-3 test beams in the second stage is used to indicate the stiffness degradation degree of different test beams. The results are listed in Table 7.

According to the result in Table 7, in the second stage, the deflection growth rate of XP-1 and XP-2 test beams is large, 39.8% and 48.64%, respectively, but the deflection growth rate of XP-3 is 6.6%, which is far less than that of other test beams. In combination with the previous test phenomena, under the corrosion–fatigue interaction, the corrosion products are mainly generated at the cracks, especially the midspan cracks. With the increase in the corrosion time, the excessive corrosion products of XP-1 and XP-2 test beams cause the concrete expansion, and the fatigue cycle further aggravates the development of this phenomenon, greatly reducing the bond between the reinforcement and the concrete. The stiffness degradation of the corrosion–fatigue coupled beam is obvious in the second stage. However, under the action of high-stress amplitude, the fatigue damage of the XP-3 test beam accumulates too quickly and occurs in a short time, so the corrosion time is short, the corrosion products are fewer, the reinforcement damage by corrosion is obviously smaller than that by the fatigue load, which results in no obvious increase in the deflection of XP-3 test beam in the second stage.

**Figure 23:**
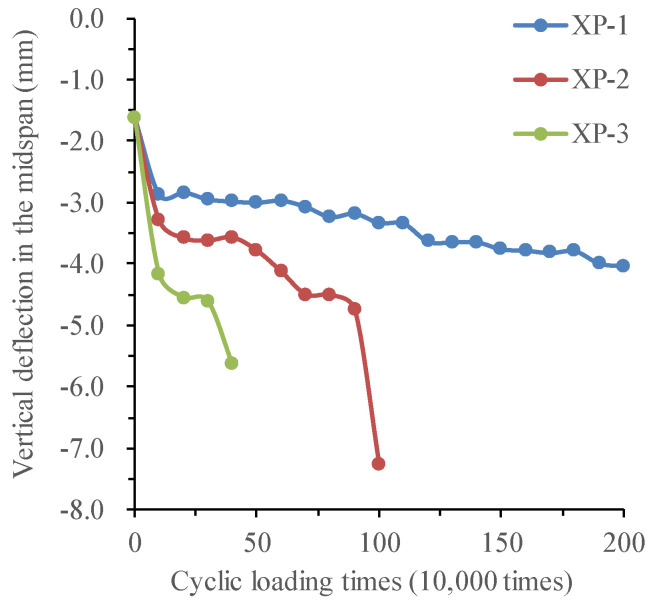
Midspan deflection of corrosion–fatigue coupling test beams under 70 kN static load.

In order to compare the stiffness degradation of the corrosion–fatigue coupling beam under different stress amplitudes, the deflection curve of each test beam with the number of cycles under 70 kN static load is plotted in Figure 23. It can be seen from this figure that when the fatigue cycle times reach 300,000, the deflection of XP-3 and XP-2 test beams is increased by 22.8% and 44.8%, respectively, compared with the XP-1 test beam. Under the condition of accelerated corrosion at the same current density and the same fatigue cycle times, the stiffness degradation amplitude of each test beam increases with the increase in stress amplitude.

(2) Mechanism analysis of the load-deflection behavior

To sum up, under the cyclic load, the stiffness of the beam is not steadily degraded gradually, and its change trend is related to the magnitude of the load. Under the fatigue load corresponding to the China Highway-I vehicle load, the stiffness of the beam body degrades obviously at the initial stage of loading, and then it is in elastic working state. After exceeding the fatigue load corresponding to the China Highway-I vehicle load by 15%, the stiffness of the test beam decreases significantly with the increase in the number of load cycles, and it is irrecoverable, which proves that irreversible damage has occurred to the beam.

The deflection growth curves of corrosion–fatigue coupling test beams are steeper than that of simple fatigue test beams, indicating that the corrosive environment accelerates the accumulation and development of fatigue damage. This is mainly due to the reduction of effective cross-section area caused by reinforcement corrosion, deterioration of reinforcement properties, and deterioration of bond performance between reinforcement and concrete caused by rust expansion cracks and corrosion products. The fatigue load undoubtedly accelerates the degradation of bond strength between corroded reinforcement and concrete, further reducing the cooperative performance of both, the tensile concrete between cracks gradually withdrawing from work, and the non-uniform coefficient of strain of longitudinal tensile reinforcement between cracks increased. Therefore, the effect of fatigue load on reducing the bearing capacity, stiffness, plastic deformation, and ductility of corroded reinforced concrete beams should be paid enough attention to.

#### 3.3.6. Fatigue Life Analysis of Test Beams

According to the upper limit of fatigue load corresponding to China Highway-I vehicle load, the fatigue life of the simple fatigue beam and corrosion–fatigue coupling beam exceeds 2 million times, and then the static load is applied to the failure. When the China Highway-I vehicle load is overloaded by 15% and 30%, the upper limit of fatigue load is 80 kN and 90 kN, respectively, and the lower limit of fatigue load remains unchanged at 20 kN. The failure state of the four overloaded test beams is fatigue failure, and the failure mode is a brittle fracture along the main crack of the section near the midspan of the beam. There is no obvious sign before the beam body is damaged. Subsequently, it is observed that the reinforcement at the bottom of the beam is broken. The fatigue failure of the four test beams occurred within 2 million cycles, and the fatigue life is listed in Table 8.

It can be seen from Table 8 that under the same stress amplitude, the fatigue life of the corrosion–fatigue coupling beam will be reduced compared with that of the simple fatigue test beam. Among them, the fatigue life of the corrosion–fatigue coupling beam XP-2 is reduced by 26.9% compared with that of the P-2 test beam; XP-3 is 18.1% less than P-3. It can be seen from the above test phenomena that the fatigue failure of the beam is mainly the fracture brittle failure of the reinforcement. Therefore, the fatigue life of the reinforced concrete beam mainly focuses on the analysis of the fatigue life of the reinforcement. The increase in the stress amplitude will obviously accelerate the expansion of the fatigue crack of the reinforcement.

## 4. Conclusions

Through the innovative design of the corrosion–fatigue coupling test loading device, the reinforcement corrosion and fatigue cyclic loading are realized simultaneously, and the influence of corrosion and fatigue on the mechanical properties of reinforced concrete T-beams is explored. Six pieces of reinforced concrete T-shaped test beams are designed and manufactured, and the simple fatigue test and corrosion–fatigue coupling test with the upper limit of fatigue load 70 kN, 80 kN, and 90 kN are carried out, respectively. The crack change and failure mode of the test beams are observed during the loading process, the strain change of longitudinal steel bars and midspan deflection are measured, and the fatigue life analysis is carried out. The following conclusions can be drawn:

(1) The designed corrosion–fatigue coupled loading device can well realize the joint action of reinforcement corrosion and fatigue loading, which can simulate the corrosion environment and the cyclic loading simultaneously with the cooperation of the spray facilities, the reinforcement accelerated corrosion device, and the loading equipment. Moreover, by observing the corrosion state of the reinforcement of the test beam, the corrosion distribution of the reinforcement presents a “point rust” shape, and the corrosion parts are concentrated in the cracks. The reinforcement at the non-cracks has no obvious corrosion traces, which is close to the corrosion situation of the reinforcement in the natural environment.
(2) For the corrosion–fatigue coupling test beams XP-1, XP-2, and XP-3, the P-1 test beam does not undergo fatigue damage when the loading times reach two million times, while the P-2 and P-3 test beams undergo fatigue damage. The number of cracks in the test beams with fatigue failure is relatively consistent. The number of cracks in the test beams without fatigue failure is significantly higher than that in the test beams with fatigue failure, the cracks are more densely distributed, and the damaged area is larger.
(3) Under the coupling action of corrosion and fatigue, the fatigue failure mode of XP-2 and XP-3 test beams is the fatigue fracture of the main reinforcement during fatigue loading. The reinforcement fracture presents a tear-inclined section shape with an uneven surface and strong grain sense, and the corrosion traces on the surface and the fracture section are serious. However, in the simple fatigue test beams P-2 and P-3, when the fatigue failure occurs, the tensile longitudinal steel bars at the bottom are broken with lamellar tearing. Although the failure mode of the corrosion–fatigue coupled test beam and the simple fatigue test beam are both fatigue failures, the fracture forms and characteristics of the reinforcement are different.
(4) Under the coupling action of corrosion and fatigue, the changes in reinforcement strain and deflection of XP-2 and XP-3 test beams show a three-stage development law similar to those of simple fatigue test beams. However, in the second stage, the reinforcement strain and deflection of simple fatigue test beams are in a stable and small growth stage, and the growth rate is lower than that of the corrosion–fatigue coupling test beam.
(5) Considering the coupling action of corrosion and fatigue, these two factors play a mutual role in promoting the fatigue crack expansion of reinforcements. In short, fatigue loading produces micro-cracks, which form permeation channels for chloride ions, oxygen, and water; accelerate the corrosion of steel bars; reduce the fatigue strength of steel bars; and the corrosion products expand the width of cracks. The whole process forms a vicious circle. At the same load amplitude, the fatigue life of the corrosion–fatigue coupling test beam is significantly reduced compared with that of the simple fatigue test beam.

## Figures and Tables

**Table 1:** Test cases.

Number of Test Beams	Lower Limit of Fatigue Load/kN	Upper Limit of Fatigue Load/kN	Stress Amplitude/MPa
XP-1	20	70	163
XP-2		80	211
XP-3		90	235
P-1	20	70	163
P-2		80	211
P-3		90	235

**Table 2:** Test phenomenon.

Item	Loading Times	Test Phenomenon
Corrosion–fatigue coupling tests	From 1 to 100,000 times	The existing cracks slowly expand, and a small number of new cracks still appear on the beam body, and a small amount of rust liquid seeps out on the surface.
	100,000 times	Most of the cracks in the test beam body have been generated. The cracks open and close repeatedly with the load, and the height of most cracks reaches the lower end of the flange.
	Fatigue failure	Its wet area is basically covered by rust when the XP-2 and XP-3 test beams have fatigue failure, but the XP-1 test beam did not have fatigue failure when it was loaded to 2 million cycles.
Simple fatigue tests	From 1 to 100,000 times	The development of the cracks is similar to the coupling test beams. They are typical bending cracks, and there are basically no inclined cracks.
	100,000 times	The development of the cracks is similar to the coupling test beams, but the width of the new cracks is relatively small, and most of them are closed when unloaded to the lower limit of the load.
	After 100,000 times	The P-1 test beam did not undergo fatigue failure when it was loaded to 2 million cycles, then the test beam was statically loaded to failure, with yield load of 160 kN and ultimate load of 252 kN.
	Fatigue failure	Both P-2 and P-3 test beams have fatigue failure before two million cycles, which is mainly manifested as brittle failure of bottom reinforcements.

**Table 3:** Fracture development law for test beams.

No. of Test Beams	Loading Stage	Fracture Development Law	No. of Test Beams	Loading Stage	Fracture Development Law
XP-1	Loaded to about 60 kN	The beam body gives a cracking “creaking” sound, and cracks appear in the midspan.	P-1	Loaded to about 68 kN	The beam body is cracked, and cracks appear on both sides of the mid-span of the test beam. There are two cracks in total.
	Loading 100,000 times	The number of cracks increases from 3 under static load to 5 under fatigue load. After the load is removed, the cracks in the mid-span are visible to the naked eye.		Loading 100,000 times	The cracks of the test beam are almost all generated, with a total of 6 cracks. The cracks that appear during static load further develop and eventually extend below the flange.
	Loading 1.2 million times	New cracks appear at both sides near the midspan at both ends of the test beam.		Loading 400,000 times	All the cracks have extended below the flange. Up to 2 million times of fatigue loading, no new cracks are generated in P-1 test beam.
	Loading 1.5 million times	The inclined crack appeared at one end of the test beam, and the average width of the inclined crack was 0.4 mm.		Loading 2 million times	The width of cracks in the midspan is 0.05 mm, and the average width of previous cracks on both sides of the midspan is 0.1 mm. The test beam has no fatigue failure.
	Loading 2 million times	The test beam has no fatigue failure.			
XP-2	Loaded to about 60 kN	It is the same as XP-1 test beam. When the load is up to the upper limit of fatigue load 80 kN, there are 4 cracks in total, most of which extend vertically along the beam height.	P-2	Loaded to about 55 kN	The beam body is cracked, and cracks appear on both sides of the midspan of the test beam. There are five cracks in total when the static load is up to the upper limit of fatigue.
	Loading 400,000 times	The crack continues to develop, and new cracks appear at both ends of the test beam.		Loading 100,000 times	The cracks are basically all generated, with a total of 8 cracks. The cracks that appear during static load further develop and finally extend below the flange.
	Loading 500,000 times	Cracks begin to appear at the flange.		Loading 500,000 times	The crack height on both sides of the midspan increases.
	Loading 1 million times	The main cracks have been extended and widened, and a small part of the concrete has fallen off.		Loading 900,000 times	The flange began to crack.
	Loading 1.01 million times	The fatigue failure occurs.		Loading 1.3 million times	Many secondary cracks have been generated around the midspan cracks.
				Loading 1.38 million times	The fatigue failure finally occurs.
XP-3	Loaded to about 60 kN	It is the same as XP-1 and XP-2 test beams. When the load is up to the upper limit of fatigue load 90 kN, there are 5 cracks in total, and they are typical bending cracks.	P-3	Loaded to about 65 kN	The beam body is cracked, and the cracks appear first in the middle span of the test beam, then cracks appear successively on both sides of the midspan. There are seven cracks in total.
	Loading 200,000 times	The crack continues to develop, and two auxiliary cracks appear near the two sides of the main crack, and new cracks appear at 0.5 m from the beam end.		Loading 100,000 times	The cracks are basically all generated, with a total of 9 cracks.
	Loading 300,000 times	Cracks appear on the flange, and some rust is found.		Loading 300,000 times	The flange of P-3 test beam is cracked.
	Loading 400,000 times	The crack on the flange develops into a branch shape, and the width of the main crack in the midspan increases.		Loading 500,000 times	The crack at the flange develops into branch-like shape, and the width of the main crack in the mid-span increase.
	Loading 430,000 times	The fatigue failure occurs.		Loading 530,000 times	The fatigue failure occurs.

**Table 4:** Failure mode of test beams.

Item	Failure Mode
Corrosion–fatigue coupling test beams	➢ The fatigue life of XP-1 test beam is more than 2 million times. The XP-2 and XP-3 test beams suffered fatigue failure before 2 million fatigue cycles. ➢ The fatigue failure forms of XP-2 and XP-3 test beams are the fatigue fracture of the main reinforcement. ➢ The fracture surface of the reinforcement presents a tear-inclined cross-sectional shape, the surface is uneven, the grain sense is strong, and the rust on the surface and the fracture section of the reinforcement are serious. ➢ The corrosion distribution of the reinforcement presents a “point rust” shape, and the corrosion is concentrated at the crack. This corrosion distribution is relatively close to the natural corrosion state of the reinforcement [37].
Simple fatigue test beams	➢ The fatigue life of P-1 test beam is more than 2 million times. The P-2 and P-3 test beams suffered fatigue failure before 2 million fatigue cycles. ➢ For the P-2 and P-3 test beams, during fatigue failure, four tensile longitudinal steel bars at the bottom two layers are broken. The concrete in the compression area rises and falls off, and the stirrups and the bottom longitudinal steel bars are exposed. ➢ The ultimate failure is caused by the fatigue brittle fracture of the tensile main reinforcement, and its failure section is perpendicular to the longitudinal direction of the reinforcement. ➢ The fatigue fracture sections of P-2 and P-3 test beams are all located in the main crack section in the pure bending part. The cross-section of the steel bar fracture is obviously divided into two parts, where one part is crescent shaped, with dim color and strong sense of grain, and another part is bright and clean with arc-shaped layered texture.

**Table 5:** Variation of longitudinal reinforcement strain in test beams.

No. of Test Beams	Longitudinal Reinforcement Strain
XP-1	➢ The tensile strain of the reinforcement in the midspan shows two stages, i.e., sudden increase and stable development.
XP-2 and XP-3	➢ The strain of the reinforcement in the midspan shows three stages, i.e., sudden increase–stable development–sudden increase. ➢ In the first stage, i.e., the number of cyclic loadings is less than 100,000 times, the strain of the reinforcement in the midspan increases rapidly. In the second stage, the strain of the reinforcement increases slowly from 100,000 cycles to near fatigue failure. In the third stage, before fatigue failure, the strain of the reinforcement in the midspan increases rapidly again. ➢ The reinforcement strain of XP-2 test beam is relatively stable before the fatigue cycle is 500,000 times, but the reinforcement strain of XP-2 test beam shows a linear growth from 500,000 cycles to near fatigue failure. ➢ The reinforcement strain of XP-3 test beam is no obvious change in the second stage of fatigue.
P-1	➢ The maximum tensile strain of the reinforcement in the midspan is 1302 με in stable stage. The reinforcement strain changes little with the increase in cyclic loading times, and the steel bar does not reach the yield strain during the whole test process.
P-2 and P-3	➢ For P-2 and P-3 test beams, the maximum tensile strain of longitudinal reinforcement adjacent to the midspan section is 1422 and 1656 με, respectively, in stable stage. ➢ The strain development experiences three stages, i.e., sudden increase, stable and accelerated increase. ➢ In the first stage, i.e., the number of cyclic loadings is less than 100,000 times, and the strain growth rate of the reinforcement is fast. In the second stage, i.e., the number of cycles is from 100,000 times to 90% of the fatigue life of the test beam, and the growth rate of the reinforcement strain is low. In the third stage, i.e., the number of cycles is greater than 90% of the fatigue life of the test beam, the strain of the reinforcement near the failure state has obvious increase, and the test beam immediately reaches failure. ➢ Until the fatigue fracture of the reinforcement occurs, the maximum strain in the reinforcement does not reach the yield strain.

**Table 6:** Variation of deflection of test beams.

No. of Test Beams	Changing Law of Midspan Deflection
XP-1	➢ The XP-1 test beam only shows the first two stages because of no fatigue failure before 2 million cycles.
XP-2 and XP-3	➢ The deflection curve presents a three-stage changing law. ➢ In the first stage, i.e., the number of cyclic loadings is less than 100,000 times, the deflection increases rapidly and gradually enters the stable period. In the second stage, i.e., from 100,000 cycles to near fatigue failure, the deflection growth rate is low, and it is in a stable development stage. In the third stage, during fatigue failure, the deflection increases rapidly again until the brittle failure of the reinforcement. ➢ In the second stage, the deflection growth rate for corrosion–fatigue coupled beams is greater than that for simple fatigue beams under the same static load.
P-1	➢ The P-1 test beam cannot be damaged after 2 million cycles. The midspan deflection of the beam in the second stage remains basically unchanged.
P-2 and P-3	➢ Similar to the corrosion–fatigue coupled beams, the deflection development of the simple fatigue beams also presents a three-stage changing law. ➢ In the first stage, i.e., the number of cyclic loadings is less than 100,000 times, the deflection increases rapidly and gradually enters the stable period. In the second stage, i.e., the number of cyclic loadings is 100,000 to near fatigue failure, the deflection growth rate is low and it is in the stable development stage. In the third stage, the deflection of the test beams near the failure state increases rapidly.

**Table 7:** Increase the value of midspan deflection of corrosion–fatigue test beams.

No. of Test Beams	XP-1	XP-2	XP-3
Deflection difference in/mm	1.16	1.80	0.27
Increase percentage of deflection	39.8%	48.64%	6.6%

**Table 8:** Fatigue life of test beams.

No. of Test Beams	Upper Limit of Fatigue Load/kN	Fatigue Life/Ten Thousand Times
P-1	70 kN	>200
XP-1		>200
P-2	80 kN	138.2
XP-2		101
P-3	90 kN	53.1
XP-3		43.5

## Data Availability

The data in this article are available from the corresponding author upon reasonable request.
